# Supplementary material for: Prognostic refinement of NSMP high-risk endometrial cancers using oestrogen receptor immunohistochemistry
Source: Br J Cancer. 2023 Jan 23;128(7):1360–8. doi: 10.1038/s41416-023-02141-0 (PMC10050005; doi:10.1038/s41416-023-02141-0)
Supplement: Supplementary file 1 — Data Supplement [file 41416_2023_2141_MOESM1_ESM.pdf]

Data Supplement

Table of contents

Supplementary material and methods

Immunohistochemistry staining procedures…………………………………………….....3

Immunohistochemistry staining scoring…………………………………………………...3

DNA isolation and sequencing………………………………………………………………4

Supplementary figures

Supplementary Figure S1. Flowchart of patient selection……………………………….5

Supplementary Figure S2. Locoregional, distant and overall recurrence-free survival, and cancer-specific survival for patients with high-risk endometrial cancer…………...6

Supplementary Figure S3. Cohort-specific recurrence-free survival by molecular subgroup……………………………………………………………………..……....7

Supplementary Figure S4. Recurrence-free survival by histologic subtype and FIGO grade in patients with MMRd, p53abn and NSMP EC……………………………………8

Supplementary Figure S5. Recurrence-free survival of NSMP EC patients by % of ER protein expression..………………………………………………………………………9

Supplementary Figure S6. Recurrence-free survival of NSMP EC patients by ER and received adjuvant treatment………………………………………………………….....10

Supplementary tables

Supplementary Table S1. Clinicopathological characteristics, by inclusion or not in the translational study…………………………………………………………………........11

Supplementary Table S2. Univariable analysis of recurrence-free survival for MMRd, NSMP and p53abn EC……………………………………………………………..………12

Supplementary Table S3. Internal validation of prognostic value of ER in NSMP EC patients………………………………………………………………………………………13

Supplementary Table S4. Multivariable analysis of recurrence-free survival including the molecular classifier with NSMP divided into ER-positive and ER-negative…………………………………………………………...……………...……14

Supplementary Notes 15

References 16

Supplementary material and methods

Immunohistochemistry staining procedures

For each case, one representative formalin-fixed paraffin-embedded tumour block was selected by a pathologist during central pathology review. Immunohistochemistry was performed on 4 µm whole slides. Slides were deparaffinized and rehydrated via graded ethanol series, followed by endogenic peroxidase activity blocking (0.3% Methanol/H_2O_2) and antigen retrieval using a microwave oven procedure in 10 mmol/L Tris-EDTA buffer, pH9.0 for 10 minutes. Tissue sections were incubated overnight with primary antibodies against p53 (clone DO-7, 1:2000, DAKO), MLH1 (clone ES05, 1:100, DAKO), MSH2 (clone FE11, 1:100, DAKO), MSH6 (clone EPR3945, 1:800, GENE TEX), ER (clone EP1, 1:400, DAKO), PR (clone PgR636, 1:200, DAKO), and L1CAM (clone 14.10, 1:800, BioLegend) at room temperature or with primary antibody PMS2 (clone EP51, 1:50, DAKO) at 4 degrees. A linker (mouse linker, SM804, DAKO; rabbit linker, SM805, DAKO) was used afterwards for MLH1, PMS2, MSH2 and MSH6. A 30 minute incubation with a secondary antibody (Poly-HRP-GAM/R/R; DPV0110HRP; ImmunoLogic) was then performed. DAB+ (K3468, DAKO) was used as chromogen and sections were counterstained with haematoxylin.

Immunohistochemistry staining scoring

For PORTEC-3 cases, MLH1, PMS2, MSH6 and MSH2 protein expression was evaluated to determine MMR status. Tumours were considered MMR deficient if more than 10% of the tumoral nuclei were negative, in the presence of a positive internal control, in at least one of the MMR proteins. For MST cases, immunohistochemistry staining of MMR proteins was performed in a two-stepped approach. Cases with more than 10% loss of PMS2 and/or MSH6 expression were considered MMR proficient. For cases with retained expression of PMS2 and MSH6, additional MLH1 and MSH2 immunohistochemistry was performed to determine final MMR status. Immunohistochemistry for p53 was considered abnormal if more than 10% of the tumour showed strong positive staining of tumour nuclei (overexpression), complete absence of staining with a positive internal control (null-mutant), or significant cytoplasmic staining (cytoplasmic)[1,2]. Immunohistochemistry for ER was considered positive if more than 10% of the tumour showed positive nuclear staining. The cut-off was chosen as it is most commonly used in the assessment of ER expression in endometrial cancer[3-6]. Finally, immunohistochemistry for PR and L1CAM were considered positive when more than 10% of the tumour showed positive nuclear staining. The same as for ER, these cut-off were chosen as they are most commonly used in EC[3,4,7,8]. All immunohistochemistry slides were

independently scored by at least two observers (TB, AL, LV). Discrepant results were resolved at simultaneous viewing.

DNA isolation and sequencing

Tumour DNA was enriched by taking three 0.8 mm tumoral tissue cores or by microdissection using 5-10 (10) slides on selected tumoral areas by a pathologist, obtaining a tumour percentage >70%. DNA isolation was performed automated using the Tissue Preparation System (Siemens Healthcare Diagnostics). After isolation, the DNA concentration was measured using a fluorometer (Qubit dsDNA HS, Life Technologies, Carlsbad, California, USA). DNA samples were sequenced using the AmpliSeq Cancer Hotspot Panel (Thermo Fisher Scientific, Waltham, MA) version 5 (PORTEC-3) and version 6 (MST). These panels are designed to detect somatic cancer hotspot mutations covering 82 genes, including the complete *POLE* exonuclease domain. Libraries were prepared using 42-84ng of DNA and each sample labelled with a unique barcode (IonCode, TheremoFisher). Ion 540 chips were prepared using Ion Chef System and sequenced using the Ion S5 sequencing System. Sequencing results were evaluated blinded for patient outcome. A minimum coverage threshold of 100 reads and a minimum variant allele frequency of 0.1 reads were considered.

Supplementary Figure S1. Flowchart of patient selection.

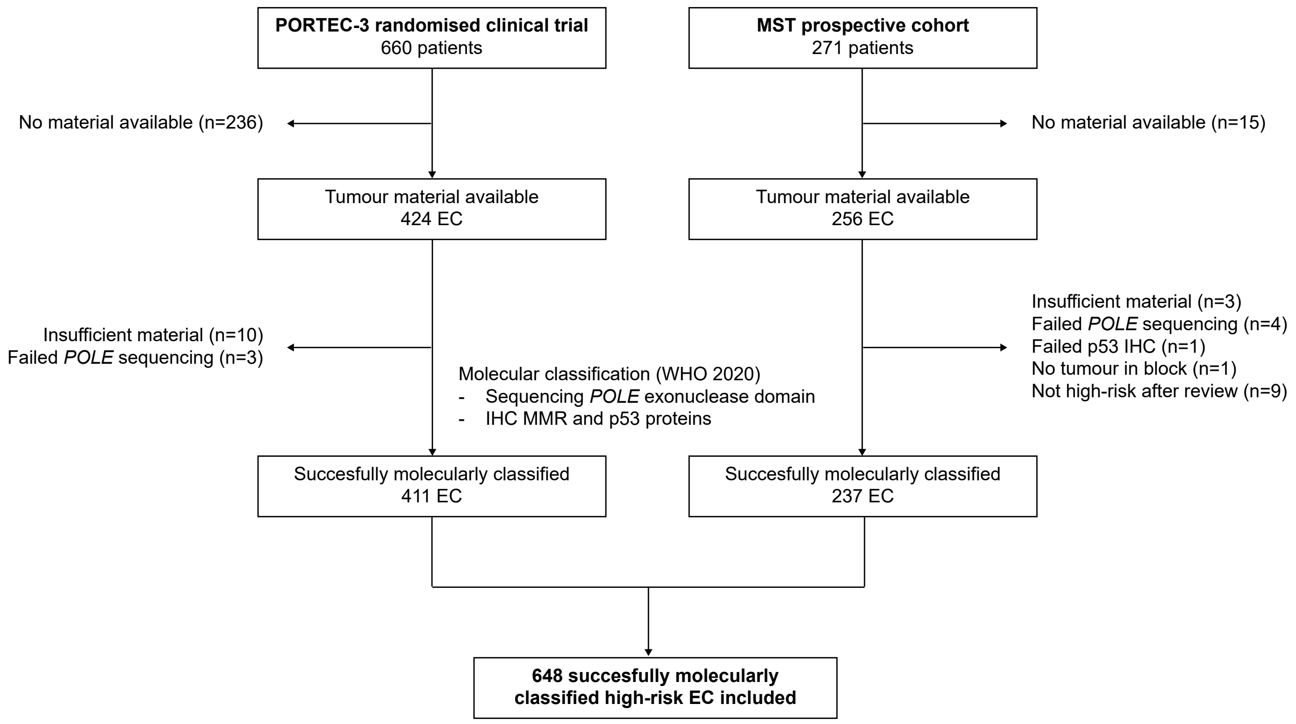

Abbreviations: EC, endometrial cancer; IHC, immunohistochemistry; WHO, World Health Organization; MMR, mismatch repair; HREC, high-risk endometrial cancer.

Supplementary figure S2. Locoregional, distant and overall recurrence-free survival, and cancer-specific survival for patients with high-risk endometrial cancer (n = 647). Kaplan-Meier survival curves of patients with high-risk endometrial cancer (EC) for (A) locoregional recurrence-free survival (RFS) for patients with *POLE*mut EC (5-year RFS 98.5%), MMRd EC (5-year RFS 88.4%), p53abn EC (5-year RFS 83.0%) and NSMP EC (5-year RFS 93.3%), (B) distant RFS for patients with *POLE*mut EC (5-year RFS 98.5%), MMRd EC (5-year RFS 73.5%), p53abn EC (5-year RFS 48.8%) and NSMP EC (5-year RFS 74.9%), (C) overall RFS for patients with *POLE*mut EC (5-year RFS 98.5%), MMRd EC (5-year RFS 71.4%), p53abn EC (5-year RFS 48.1%) and NSMP EC (5-year RFS 74.5%), and (D) cancer-specific survival for patients with *POLE*mut EC (5-year RFS 98.5%), MMRd EC (5-year RFS 79.1%), p53abn EC (5-year RFS 54.2%), NSMP EC (5-year RFS 84.8%).

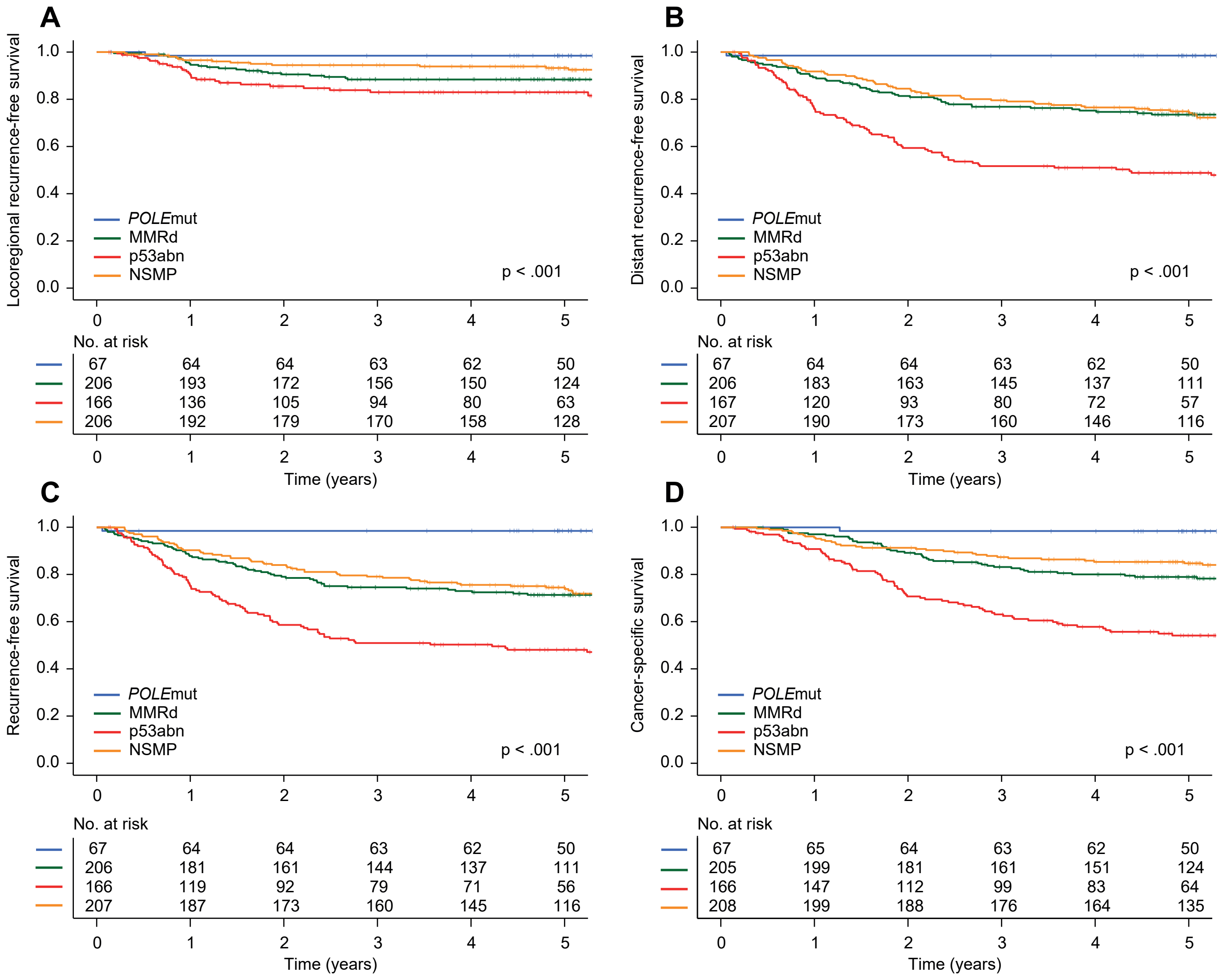

Abbreviations: *POLE*mut, *POLE*-ultramutated; MMRd, mismatch repair deficient; p53abn, p53-abnormal; NSMP, no specific molecular profile.

Supplementary Figure S3. Cohort-specific recurrence-free survival by molecular subgroup. Kaplan-Meier survival curves of patients with high-risk endometrial cancer from (A) PORTEC-3 and (B) MST for recurrence-free survival by molecular subgroup.

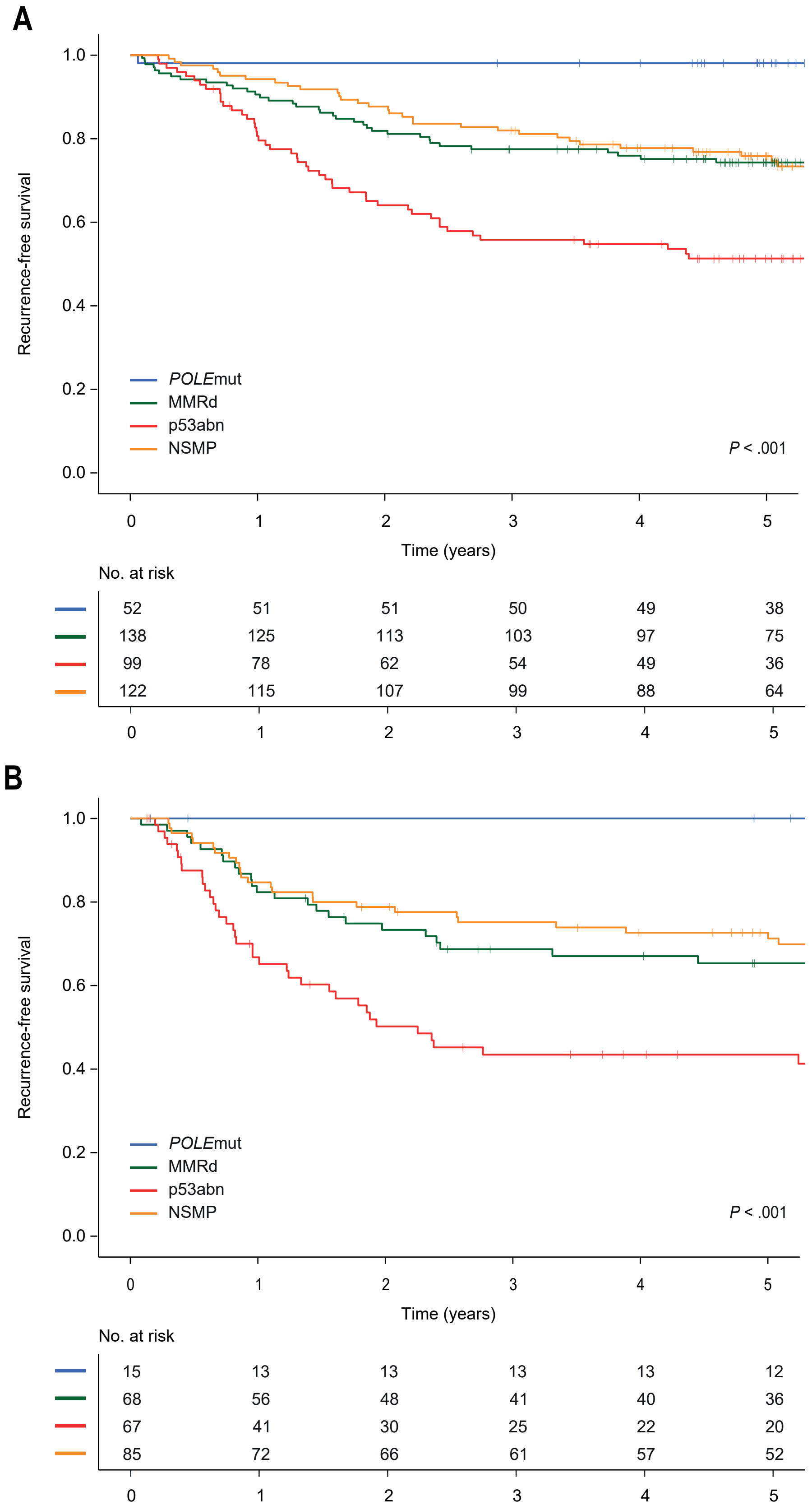

Supplementary Figure S4. Recurrence-free survival by histologic subtype and FIGO grade. Kaplan-Meier survival curves of (A) mismatch repair-deficient (MMRd), (B) p53-abnormal (p53abn) and (C) no specific molecular profile (NSMP) high-risk endometrioid endometrial cancer (EEC) for recurrence-free survival by histologic subtype and FIGO grade.

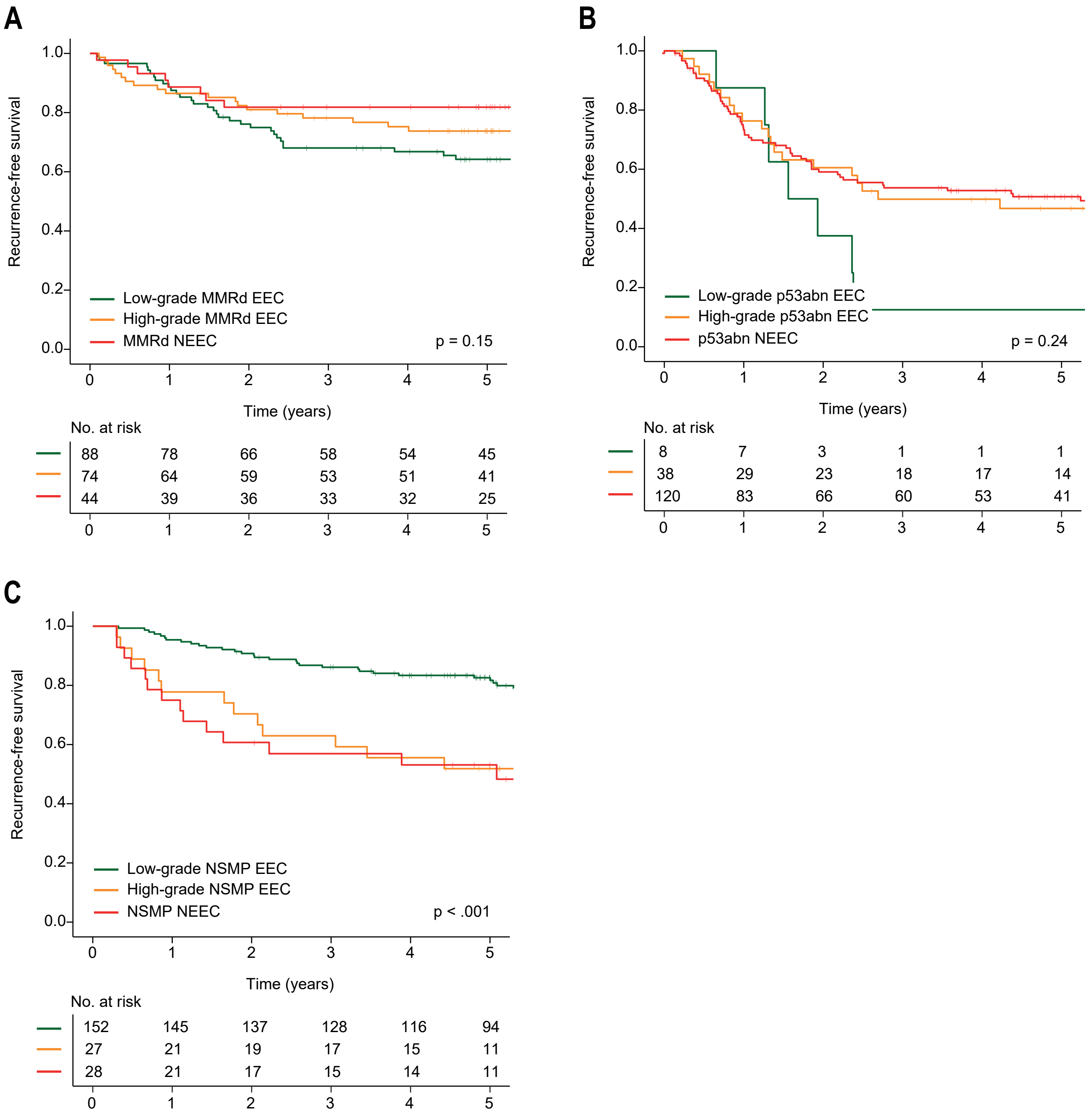

Supplementary Figure S5. Recurrence-free survival by % of ER protein expression.
Kaplan-Meier survival curves of patients with NSMP high-risk endometrial cancers for recurrence-free survival by different levels of tumour ER protein expression.

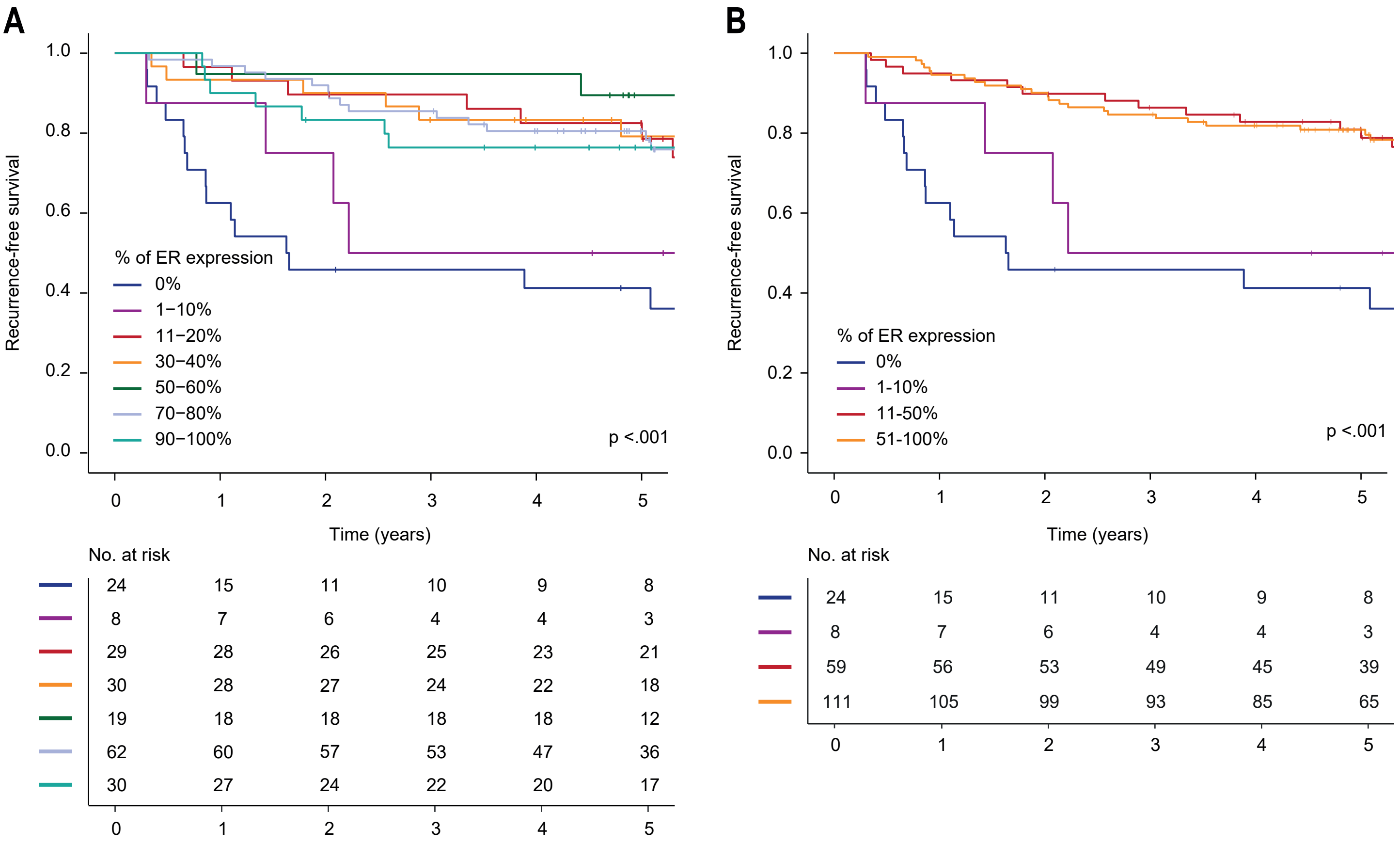

Supplementary Figure S6. Recurrence-free survival of NSMP endometrial cancer patients by ER status and received adjuvant treatment. Kaplan-Meier survival curves of patients with NSMP high-risk endometrial cancer for recurrence-free survival by ER status and received adjuvant treatment.

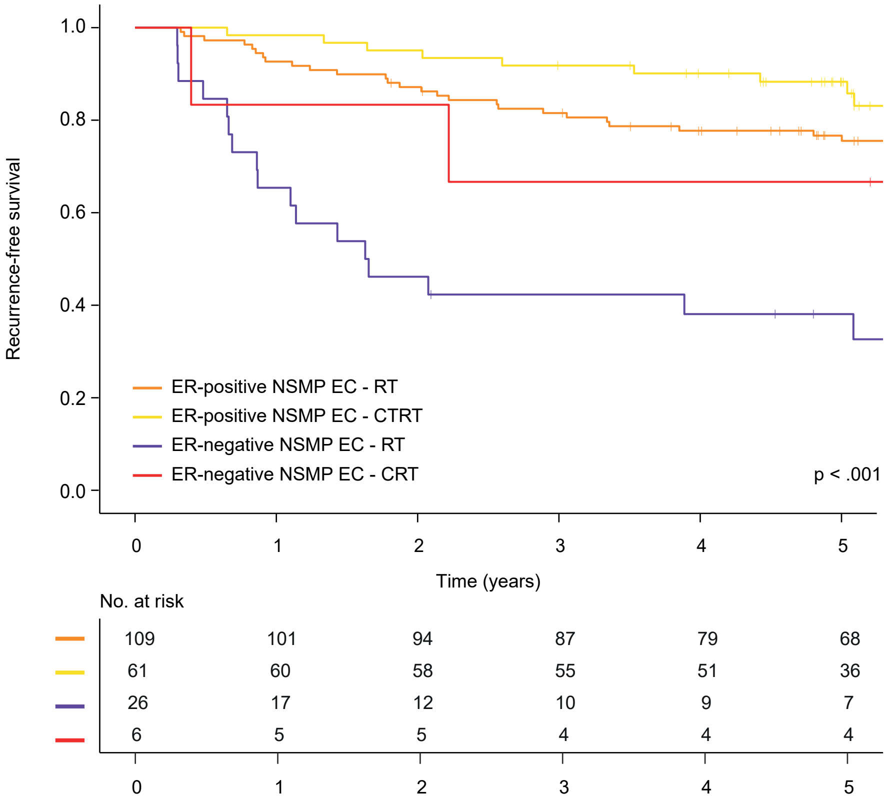

Supplementary Table S1. Clinicopathological characteristics, by inclusion or not in the translational study.

	**Included**<br>n = 648 (100.0%)	**Excluded**<br>n = 257 (100.0%)	**Total**<br>n = 905 (100.0%)	*p*-value
Age				0.16
Mean (range)	63.8 (25.0-92.0)	62.8 (36.1-62.8)	63.6 (25.0-92.0)	
Histotype and grade				0.27
Low-grade endometrioid	254 (39.2)	96 (37.4)	350 (38.7)	
High-grade endometrioid	179 (27.6)	74 (28.8)	253 (28.0)	
Serous	88 (13.6)	42 (16.3)	130 (14.4)	
Clear cell	53 (8.2)	22 (8.6)	75 (8.3)	
Mixed	31 (4.8)	14 (5.4)	45 (5.0)	
Carcinosarcoma	24 (3.7)	1 (0.4)	25 (2.8)	
Un-/dedifferentiated	16 (2.5)	7 (2.7)	23 (2.5)	
Other	3 (0.5)	1 (0.4)	4 (0.4)	
Stage				0.11
IA	76 (11.7)	26 (10.1)	102 (11.3)	
IB	131 (20.2)	45 (17.5)	176 (19.4)	
II	181 (27.9)	68 (26.5)	249 (27.5)	
III	260 (40.1)	118 (45.9)	378 (41.8)	
LVSI				0.16
Absent	341 (52.6)	122 (47.5)	463 (51.2)	
Present	307 (47.4)	135 (52.5)	442 (48.8)	
Received treatment				0.001
EBRT	403 (62.5)	133 (51.8)	536 (59.4)	
EBRT + CT*	223 (34.6)	121 (47.1)	344 (38.1)	
VBT	19 (2.9)	3 (1.2)	22 (2.4)	
Follow-up time (years)				<.001
Median (95% CI)	7.0 (6.7-7.2)	6.1 (5.9-6.4)	6.6 (6.3-6.9)	
Overall survival				0.031
5-year estimate	71.7%	77.0%	73.2%	

* Including two patients who received VBT+CT.

Abbreviations: LVSI, lymphovascular space invasion; EBRT, external beam radiotherapy; CT, chemotherapy; VBT, vaginal brachytherapy.

Supplementary Table S2. Univariable analysis of overall recurrence-free survival for MMRd, NSMP and p53abn EC.

		MMRd EC				NSMP EC				p53abn EC			
		Total n	HR	95% CI	*p*-value	Total n	HR	95% CI	*p*-value	Total n	HR	95% CI	*p*-value
Age	≤ 60 years	83	1			85	1			20	1		
	> 60 years	123	1.590	0.903-2.798	0.11	122	1.488	0.871-2.542	0.15	146	1.103	0.565-2.152	0.77
Histology and grade	EEC, low-grade	88	1			152	1			8	1		
	EEC, high-grade	74	0.705	0.398-1.248	0.23	27	2.592	1.369-4.908	0.003	38	0.573	0.243-1.355	0.21
	NEEC	44	0.507	0.232-1.107	0.09	28	2.939	1.575-5.484	0.001	120	0.538	0.244-1.187	0.13
Stage	I	60	1			25	1			81	1		
	II	63	1.799	0.800-4.049	0.16	75	0.374	0.165-0.846	0.018	32	1.821	0.980-3.384	0.06
	III	83	3.295	1.559-6.964	0.002	107	0.827	0.412-1.661	0.59	53	4.077	2.470-6.732	<.001
LVSI	Absent	97	1			123	1			86	1		
	Present	109	1.229	0.695-2.174	0.48	84	1.928	1.105-3.365	0.021	80	1.448	0.927-2.264	0.10
Treatment received	EBRT	136	1			137	1			105	1		
	EBRT+CT	69	1.116	0.601-2.072	0.73	70	0.563	0.296-1.070	0.08	59	0.675	0.402-1.133	0.14
ER IHC	Negative (<10%)	41	1			32	1			81	1		
	Positive (≥10%)	150	1.343	0.656-2.748	0.42	170	0.274	0.157-0.478	<.001	82	1.195	0.777-1.838	0.42
PR IHC	Negative (<10%)	79	1			53	1			115	1		
	Positive (≥10%)	124	1.854	1.026-3.352	0.041	149	0.395	0.234-0.668	0.001	47	0.940	0.585-1.510	0.80
L1CAM IHC	Negative (<10%)	176	1			182	1			50	1		
	Positive (≥10%)	28	0.582	0.232-1.462	0.25	24	1.856	0.929-3.709	0.08	114	1.241	0.764-2.016	0.38
CTNNB1 exon 3	No mutation	153	1			118	1			144	1		
	Mutation	19	1.018	0.402-2.579	0.97	51	0.807	0.435-1.497	0.50	0	NA		

Abbreviations: MMRd, mismatch repair-deficient; EC, endometrial cancer; NSMP, no specific molecular profile; p53abn, p53-abnormal; HR, hazard ratio; CI, confidence interval; EEC, endometrioid endometrial cancer; NEEC, non-endometrioid endometrial cancer; LVSI, lymphovascular space invasion; EBRT, external beam radiotherapy; CT, chemotherapy.

Supplementary Table S3. Internal validation of prognostic value of ER in NSMP EC patients.

	PORTEC-3 RFS (32 events)				MST RFS (28 events)			
	Total n	HR	95% CI	*p*-value	Total n	HR	95% CI	*p*-value
Histology and grade								
Endometrioid, low-grade	90	1			58	1		
Endometrioid, high-grade	13	2.10	0.78-5.70	0.14	13	2.54	0.86-7.50	0.09
Non-endometrioid	14	1.58	0.41-6.08	0.50	14	1.44	0.37-5.6	0.60
Stage								
I-II	52	1			45	1		
III	65	2.04	0.95-4.36	0.07	40	2.31	1.01-5.31	0.048
Treatment received*								
RT (VBT or EBRT)	55	1			NP			
RT (VBT or EBRT) + CT	62	0.50	0.24-1.02	0.06				
ER IHC								
Negative (<10%)	13	1			19	1		
Positive (≥10%)	104	0.31	0.09-1.06	0.06	66	0.32	0.10-1.03	0.06

* Analysis of MST patient not corrected for treatment as the majority of patients (93.1%) were treated with radiotherapy alone.

Abbreviations: RFS, recurrence-free survival; HR, hazard ratio; CI, confidence interval; RT, radiotherapy; VBT, vaginal brachytherapy; EBRT, external beam radiotherapy; CT, chemotherapy; IHC, immunohistochemistry; NP, not performed.

Supplementary Table S4. Multivariable analysis of overall recurrence-free survival including the molecular classifier with NSMP divided into ER-positive and ER-negative.

**Recurrence**	**Multivariable analysis**		
n = 643, 207 events	HR	95% CI	*p*-value
Age			
≤60 years	1		
>60 years	1.39	0.99-1.95	0.06
Stage			
I	1		
II	1.54	0.99-2.41	0.06
III	3.25	2.21-4.76	<.001
Histology and grade			
Endometrioid, low-grade	1		
Endometrioid, high-grade	1.15	0.75-1.76	0.52
Non-endometrioid	1.01	0.64-1.60	0.97
LVSI			
Absent	1		
Present	1.29	0.95-1.76	0.10
Treatment received			
RT (VBT or EBRT)	1		
RT (VBT or EBRT) + CT	0.68	0.48-0.95	0.023
Molecular subgroups			
MMRd	1		
*POLE*mut	0.12	0.03-0.50	0.003
p53abn	2.82	1.90-4.19	<.001
ER-positive NSMP	0.69	0.45-1.06	0.09
ER-negative NSMP	2.27	1.33-3.90	0.003

Model fit multivariable model: Akaike's information criterion (AIC) 2162.38, model concordance (C-index) 0.726.
Abbreviations: HR, hazard ratio; CI, confidence interval; LVSI, lymphovascular space invasion; RT, radiotherapy; VBT, vaginal brachytherapy; EBRT, external beam radiotherapy; CT, chemotherapy; MMRd, mismatch repair-deficient; *POLE*mut, *POLE* ultra-mutated; p53abn, p53-abnormal; NSMP, no specific molecular profile.

Supplementary Notes

Members of the TransPORTEC consortium

Department of Radiation Oncology N Horeweg, S M de Boer, C L Creutzberg, department of Pathology T Bosse, V T H B M Smit, and Department of Medical Oncology J Kroep, Leiden University Medical Center, Leiden, The Netherlands; Department of Radiation Oncology, Erasmus MC Cancer Center, Rotterdam, The Netherlands R A Nout; Department of Gynaecologic Oncology, University Medical Center Groningen, The Netherlands H W Nijman, de Bruyn M. Department of Clinical Oncology M E Powell and Department of Cellular Pathology N Singh, Barts Health NHS Trust, London, UK; Manchester Academic Health Science Centre, St Mary's Hospital, Obstetrics and Gynaecology, Manchester, UK H C Kitchener, E Crosbie, Edmondson R; Oxford University Hospitals NHS Foundation Trust, Oxford NIHR Comprehensive Biomedical Research Centre, Oxford, UK D N Church. Department of Radio-therapy, Institut Gustave Roussy, Villejuif, France A Leary, Division of Cancer Medicine, Peter MacCallum Cancer Centre, Melbourne, VIC, Australia L Mileshkin; School of Biomedical Sciences, Faculty of Health, Queensland University of Technology, Translational Research Institute, Princess Alexandra Hospital Campus, Brisbane, Australia P M Pollock. Odette Cancer Center, Sunnybrook Health Sciences Centre, Medical Oncology, Toronto, Canada H MacKay.

References

1 Vermij, L., Leon-Castillo, A., Singh, N., Powell, M. E., Edmondson, R. J., Genestie, C. *et al.* p53 immunohistochemistry in endometrial cancer: clinical and molecular correlates in the PORTEC-3 trial. *Mod Pathol* 10.1038/s41379-022-01102-x (2022).

2 Singh, N., Piskorz, A. M., Bosse, T., Jimenez-Linan, M., Rous, B., Brenton, J. D. *et al.* p53 immunohistochemistry is an accurate surrogate for TP53 mutational analysis in endometrial carcinoma biopsies. *J Pathol* **250**, 336-345 (2020).

3 Trovik, J., Wik, E., Werner, H. M., Krakstad, C., Helland, H., Vandenput, I. *et al.* Hormone receptor loss in endometrial carcinoma curettage predicts lymph node metastasis and poor outcome in prospective multicentre trial. *Eur J Cancer* **49**, 3431-3441 (2013).

4 van der Putten, L. J. M., Visser, N. C. M., van de Vijver, K., Santacana, M., Bronsert, P., Bulten, J. *et al.* Added Value of Estrogen Receptor, Progesterone Receptor, and L1 Cell Adhesion Molecule Expression to Histology-Based Endometrial Carcinoma Recurrence Prediction Models: An ENITEC Collaboration Study. *Int J Gynecol Cancer* **28**, 514-523 (2018).

5 Jongen, V., Briet, J., de Jong, R., ten Hoor, K., Boezen, M., van der Zee, A. *et al.* Expression of estrogen receptor-alpha and -beta and progesterone receptor-A and -B in a large cohort of patients with endometrioid endometrial cancer. *Gynecol Oncol* **112**, 537-542 (2009).

6 Mileshkin, L., Edmondson, R., O'Connell, R. L., Sjoquist, K. M., Andrews, J., Jyothirmayi, R. *et al.* Phase 2 study of anastrozole in recurrent estrogen (ER)/progesterone (PR) positive endometrial cancer: The PARAGON trial - ANZGOG 0903. *Gynecol Oncol* **154**, 29-37 (2019).

7 Stelloo, E., Nout, R. A., Osse, E. M., Jurgenliemk-Schulz, I. J., Jobsen, J. J., Lutgens, L. C. *et al.* Improved Risk Assessment by Integrating Molecular and Clinicopathological Factors in Early-stage Endometrial Cancer-Combined Analysis of the PORTEC Cohorts. *Clin Cancer Res* **22**, 4215-4224 (2016).

8 Kommoss, F. K., Karnezis, A. N., Kommoss, F., Talhouk, A., Taran, F. A., Staebler, A. *et al.* L1CAM further stratifies endometrial carcinoma patients with no specific molecular risk profile. *Br J Cancer* **119**, 480-486 (2018).
